# A Scoping Review of Exercise Oncology in the Primary Brain Tumor Patient–Caregiver Dyad

**DOI:** 10.3390/curroncol33040193

**Published:** 2026-03-30

**Authors:** Anh Huan Vo, Maximilian Libmann, David Carson, Kimberly Wang, Sushant Puri, Nicholas Butowski, Kerri Winters-Stone

**Affiliations:** 1 Department of Neurological Surgery, Oregon Health & Science University, Portland, OR 97201, USA; 2 Knight Cancer Institute, Oregon Health & Science University, Portland, OR 97239, USA; 3 Department of Biochemistry and Molecular Biology, Oregon State University, Corvallis, OR 97331, USA; 4 Oregon Health & Science University Library, Portland, OR 97239, USA; 5 Department of Neurology, Oregon Health & Science University, Portland, OR 97239, USA; 6 Department of Neurological Surgery, University of California, San Francisco, CA 94143, USA; 7 Department of Cancer Population Science, Oregon Health & Science University, Portland, OR 97239, USA

**Keywords:** primary brain tumor, CNS tumor, caregiver, dyad, exercise, physical activity

## Abstract

Background: Primary malignant brain tumors (PBT) impose substantial burdens on patients and caregivers. Caregivers are essential in the delivery of outpatient care for patients with PBT but experience high levels of fatigue, distress, and health decline. Although exercise is known to improve outcomes in cancer patients, interventions tailored specifically to the PBT patient–caregiver dyad remain limited. Dyadic intervention, as well as exercise oncology, are emerging areas of active research in neuro-oncology. This scoping review incorporates both principles to evaluate the existing literature on exercise interventions on primary brain tumor patient–caregiver dyads. Methods: We conducted a comprehensive search of MEDLINE (PubMed), Embase, CINAHL (EBSCO), Rehabilitation & Sports Medicine (EBSCO), and Cochrane Central (Ovid) in December 2025 for studies involving exercise interventions that included adult PBT patients and caregivers. Results: Of the 1126 records screened, eight studies were included: four yoga-based interventions (three feasibility trials and one ongoing multicenter RCT), one pilot ski-based intervention, and three aerobic and resistance training-based interventions (two qualitative and one ongoing trial). The interventions were safe and feasible, with high adherence and retention. The preliminary reported benefits included improvements in fatigue, sleep, quality of life, and caregiver distress for the dyads. Videoconference delivery was effective, particularly during the COVID-19 pandemic. The eight included studies comprised 5–67 dyads, with four being single-arm feasibility studies. Conclusions: Current literature on dyadic exercise intervention in neuro-oncology consists primarily of small-scale feasibility and pilot studies. Initial findings have demonstrated that such interventions are safe. However, preliminary efficacy remains limited due to the risk of bias and lack of statistical power. Larger randomized clinical trials with objective endpoints are needed to define efficacy and guide evidence-based protocols.

## Simple Summary

This scoping review examines how exercise therapy programs intended for primary brain tumor patients and their caregivers impact the quality of life of both the patient and the caregiver. Following a search of major databases, eight studies were included. Four yoga-based interventions, one ski-based intervention, and three aerobic and resistance training-based interventions were included. Although more studies are needed, early evidence indicates that dyadic exercise interventions are safe and feasible in primary brain tumor dyads.

## 1. Introduction

Primary malignant brain tumors (PBTs), most commonly glioblastoma (GBM), are among the most devastating cancers in terms of both prognosis and impact. GBM has a median overall survival of only 16–18 months despite optimal standard-of-care therapy consisting of maximal safe resection, radiotherapy, and concurrent/adjuvant temozolomide [1]. Temozolomide, an oral alkylating agent, is typically administered at home under the supervision of a caregiver, often an intimate partner, rather than in a clinical setting [2,3]. Caregivers in this role provide essential support in medication administration, symptom monitoring, and coordination of care, yet frequently experience significant emotional strain, physical burden, and deterioration in their own health alongside the patient [4,5,6,7]. Other PBT subtypes, including astrocytoma and oligodendroglioma (WHO grades 2–4), remain incurable despite more favorable prognoses (median survival 4–15 years) [1]. Standard treatment for these tumors also involves radiotherapy and oral chemotherapy: ranging from temozolomide to lomustine/procarbazine/vincristine combinations or targeted agents such as vorasidenib, placing the caregiver at the center of day-to-day treatment delivery [8]. WHO grade 1 tumors such as meningioma or pilocytic astrocytoma, although potentially curable with surgery and not included in all studies, can also manifest with challenging neurologic symptoms, creating an additional burden for the PBT patient–caregiver dyad [9].

Cancer diagnosis and treatment often lead to a decline in physical activity levels and physical functioning. Exercise is known to improve fatigue, functional outcomes, quality of life, and even survival in oncology populations [10,11,12,13,14,15,16,17]. In PBT patients, concerns about seizures, neurological deficits, treatment side effects, and disease-related impairments present distinct barriers to engagement in exercise [7,18,19,20,21,22,23,24]. Several feasibility studies have demonstrated that exercise interventions are both safe and beneficial for neuro-oncology patients and improve strength, cancer-related fatigue, and health-related quality of life [25,26,27,28,29,30]. However, these studies have focused exclusively on patients, with limited evaluation of interventions targeting the patient–caregiver dyad. Data from a recent study suggests that primary brain tumor patients would prefer to exercise alongside their spouse or caregiver [31,32].

Although considered rare diseases, PBTs account for a disproportionate share of cancer-related morbidity and mortality [33]. Their management carries a substantial economic burden due to the need for advanced imaging, neurosurgical intervention, and long-term supportive care. The patient–caregiver dyad is uniquely critical in neuro-oncology, as many patients face mobility limitations, cognitive impairment, or treatment-related fatigue that increase dependence on caregivers [9,10,11,12,13,34]. Importantly, recent evidence suggests that improving quality of life within the patient–caregiver dyad can improve PBT patient survival [35,36,37]. As the patient–caregiver dyad in PBT is an interdependent team, dyadic structured exercise intervention can potentially be more beneficial than the patient or caregiver exercising independent of one another [7,38]. At the same time, exercise oncology is a new and exciting area of active research. A recent phase three trial demonstrated that a structured exercise program can improve survival in patients with colon cancer [17]. Large randomized clinical trials including hundreds of dyads have demonstrated that exercise interventions improve quality of life for both patients and caregivers in cancer dyads, including lungs, breasts, and prostate cancers [34,39]. A systematic review also suggested that caregivers may experience additional physical and psychosocial benefits from participating in these dyadic interventions [40]. Although a previous observational study, as well as preclinical data, does support the benefits of exercise on survival in patients with a primary brain tumor, robust clinical trials demonstrating comparable benefits in the field of neuro-oncology are lacking despite the well-established evidence of exercise on brain health [41,42]. This scoping review incorporates both principles of dyadic intervention and exercise oncology to synthesize and evaluate the existing literature on exercise interventions involving primary brain tumor patient–caregiver dyads. By mapping out the current literature and examining the available studies, we aimed to characterize the range of dyadic intervention models that have been tested, and summarize their safety, feasibility, and quality of life outcomes. This scoping review will also identify gaps in current knowledge to inform the development of evidence-based exercise protocols and future randomized clinical trials in neuro-oncology for PBT dyads.

## 2. Methods

This study was conducted following the Preferred Reporting Items for Systematic reviews and Meta-Analyses extension for Scoping Reviews (PRISMA-ScR) guidelines [43]. This scoping review was registered with INPLASY (Middletown, DE, USA) (registration number: INPLASY202620011).

### 2.1. Search Methods

**Figure 1:**
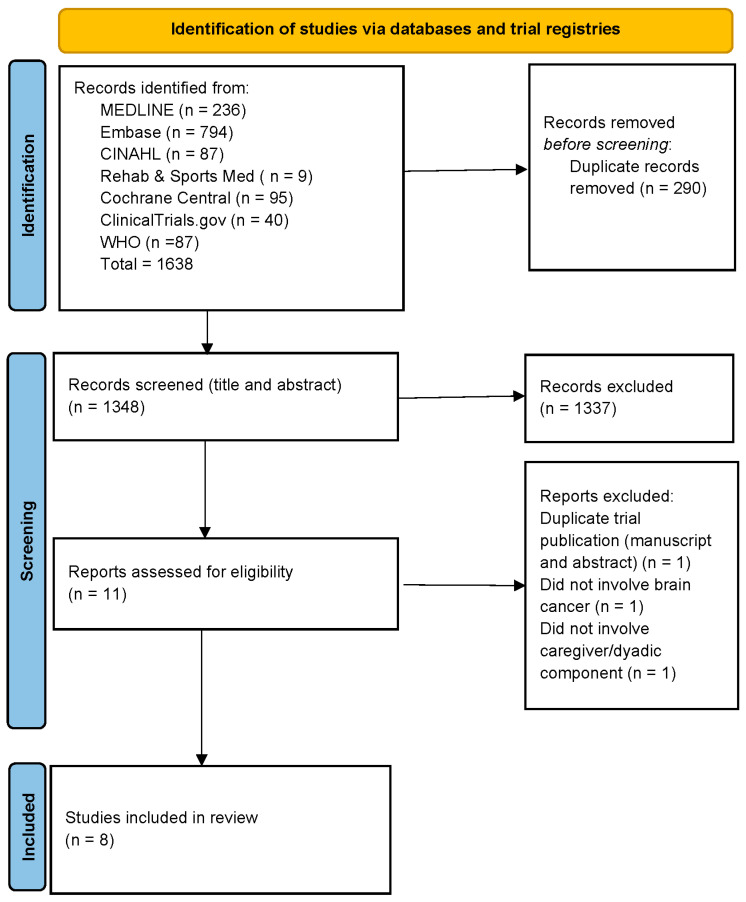
Literature search and studies included.

We conducted comprehensive searches of MEDLINE (PubMed), Embase, CINAHL (EBSCO), Rehabilitation & Sports Medicine (EBSCO), and Cochrane Central (Ovid) on 17 December 2025, including all articles available up to that date (Figure 1). ClinicalTrials.gov and the WHO International Clinical Trials Registry Platform (ICTRP) were searched for completed and ongoing trial records. We searched broadly across three main concepts: “primary brain tumor,” “caregiver,” and “exercise.” To capture all relevant articles, multiple synonyms and term variations were developed for each concept. Both index terms and keywords were incorporated into the search strategy. No publication date or language limits were applied. The full search strategies are included in Supplemental File S1. Gray literature was explored by manually reviewing the reference lists of included studies and examining conference abstracts. References were managed using Zotero.

### 2.2. Study Selection

All titles and abstracts identified in the initial database search were independently screened. Studies were included if they met the following criteria: (1) focused on adult primary brain tumors, (2) employed a dyadic intervention or involved the caregiver, and (3) incorporated an exercise oncology component such as a physical activity program. Articles and abstracts were excluded if they were systematic reviews or case studies. This scoping review aimed to include both interventional as well as observational studies to map out the current literature. To qualify, a study must include both members of the patient–caregiver dyad, although exercise interventions could be delivered concurrently (as a dyadic unit) or separately (targeting the patient but the caregiver can also participate alongside). Studies were included if reported outcomes for at least one member of the dyad were reported. Given the exploratory nature of this scoping review, there were no restrictions on the types of outcomes reported.

## 3. Results

### 3.1. Search and Selection Process

An article search was carried out as described in Figure 1, resulting in 1638 references. After removing duplicates, we had 1348 references. Subsequently, AHV and ML independently screened all 1348 records by title and abstract and included 11 references. Further review of the completed manuscript, as well as additional hand searched articles in these references, resulted in a final count of eight publications included in this review after removing duplicates and records that did not meet the inclusion criteria. The characteristics of the studies included can be found in Table 1. Measures were collected from both the patient participants and caregiver participants unless otherwise noted. The dose and description of the prescribed structured exercise programs are included in Table 2. No additional unique studies meeting the inclusion criteria were added after the gray literature search.

### 3.2. Study Description and Outcomes

The most substantial body of research on dyadic exercise interventions for PBT patients and caregivers has been conducted through yoga-based programs. Early work by Milbury and colleagues supports the feasibility of yoga as a supportive care strategy for PBT patients with high-grade glioma (HGG) undergoing radiotherapy and their caregivers in a single-arm pilot trial in 2017 [44]. The intervention was 2 or 3 weekly sessions during the 5–6-week course of radiation therapy, and was composed of joint loosening with mindfulness training, deep relaxation techniques, yoga-focused sound resonance and guided meditation. There were also components of dyadic posture, reciprocal support, and teamwork. This study approached six dyads, of which five dyads consented and completed the intervention, with all dyads reporting benefits from the program. The patient reported outcomes (PROs) included: Brief Fatigue Inventory (BFI), Center for Epidemiological Studies–Depression measures (CES-D), MD Anderson Symptom Inventory (MDASI), Study 36-item Short Form Survey (SF-36), and Pittsburgh Sleep Quality Index (PSQI). The study also demonstrated improvements in PROs, most notably in patient’s fatigue (as measured by the BFI), sleep (PSQI), and patient and caregiver mental quality of life, as measured by the mental composite summary score (MCS) domain within the SF-36 survey. Statistical analysis was performed using descriptive analyses, as well as paired sample *t*-tests. This trial established the proof-of-concept that a dyadic exercise-based intervention is both feasible, safe, and capable of addressing the multifaceted needs of patients and caregivers navigating neuro-oncology care. However, the risk of bias is high due to the largely descriptive nature of this study.

Building upon this foundation, in 2019, Milbury and colleagues expanded the evaluation of dyadic yoga with a larger feasibility randomized controlled trial with a waitlist control group involving patients with high-grade glioma and their caregivers [45]. This study refined the intervention protocol, standardizing session structure while retaining flexibility for neuro-oncology-specific limitations such as motor deficits, seizure risk, and fatigue. Twenty patient–caregiver dyads, the majority with WHO grade 4 gliomas, were randomized into a 12-session dyadic yoga intervention or waitlist control group. Patient–caregiver dyads underwent 2–3 weekly 45-min yoga intervention sessions for 12 total sessions during radiation therapy. A priori feasibility criteria were met: 70% of the dyads consented following the information visit, and 88% and 95% of the dyads demonstrated adherence and retention, respectively. Preliminary efficacy signals were measured using PROs: MD Anderson Symptom Inventory—Brain Tumor (MDASI-BT), CES-D, and BFI, SF-36. Statistical analysis was performed using descriptive analyses, as well as paired sample *t*-test and was controlled for confounding factors such as age and performance status using general linear modeling. This trial demonstrated a clinically significant reduction in overall cancer-related symptoms, as measured by the MDASI-BT, along with meaningful improvements in mental quality of life and fatigue among patient–caregiver dyads, as measured by the MCS domain of SF-36 and the BFI. Additionally, medium effect sizes were observed in cognitive, neurological, and general disease-related symptoms. The trial provided further evidence of strong feasibility, with most dyads able to complete the prescribed sessions during active radiotherapy. This trial is limited by the lack of a control group and its small sample size.

Milbury and colleagues published the largest dyadic exercise trial to date in 2022, investigating dyadic yoga interventions for primary brain tumor patient-caregiver dyads through a three-arm randomized controlled trial comparing dyadic and individual yoga programs [46]. The yoga intervention included two to three 45-min sessions per week during radiation therapy for a total of 15 sessions. All sessions focused on Hatha Yoga, whereas the dyadic sessions also focused on mutual support, as well as dyadic postures, during practice. The trial randomized 67 dyads into dyadic and individual caregiver yoga intervention arms, as well as a usual care arm. The PROs included CES-D, SF-36, and Caregiver Reaction Assessment (CRA). They also measured program evaluation variables that were developed for their yoga trials. Descriptive statistics, as well as *t*-test and multilevel-modeling, were utilized. Interestingly, although caregivers in all groups recommended the intervention to other caregivers, caregivers in the individual caregiver yoga intervention arm perceived greater benefits than in the dyadic intervention arm, which was consistent with qualitative assessment. This was attributed to the benefits of respite, relaxation, and self-care in the individual yoga caregiver arm. There was still a benefit in mental quality of life, as measured by the MCS domain of the SF-36 survey in the dyadic intervention arm compared to the usual care arm. Of note, this trial also happened during the pandemic, which caused additional difficulty regarding enrollment, and many were lost to follow up. All sessions were delivered via Zoom, a HIPAA-compliant videoconference platform, for participants enrolled during the COVID-19 pandemic. However, the study was not adequately powered for efficacy and there was no yoga intervention arm for patients only. An adequately powered and larger trial is warranted to evaluate the efficacy of dyadic exercise interventions in the PBT patient–caregiver dyad. Importantly, this study demonstrated that the videoconference platform is feasible for the delivery of exercise interventions in the primary brain tumor patient–caregiver dyad population.

Most recently, the YINOTA-O trial protocol was published in 2024 investigating a tailored yoga intervention delivered via videoconference format for 1 h per week over an 8-week period for [47]. This was a multicenter randomized controlled trial enrolling 70 PBT patient–caregiver dyads with a waitlist control group. The primary endpoint was a change in self-reported generalized anxiety, as measured by the Generalized Anxiety Disorder-7 questionnaire (GAD-7), and secondary endpoints were patient-reported quality of life metrics, as well as biomarkers, including brain-derived neurotropic factors, dehydroepiandrosterone/dehydroepiandrosterone sulfate, ferritin, and hair cortisol. If successful, this study may provide the evidence base required for the integration of exercise-based dyadic supportive care into routine neuro-oncology practice.

Exercise interventions with a more recreational focus have also been attempted. In 2022, Troschel and colleagues in Germany published a pilot study evaluating the feasibility, safety, and short-term effects of a one-week, ski-based exercise intervention among primary brain tumor patients (WHO Grade 1–4) and their caregivers (nine patients and six family members), supplemented by the presence of 13 children who participated informally but were excluded from the formal analysis [48]. Daily ski sessions were led by professional guides, two of them board-certified physicians, and monitored by medical staff. Participants completed the program without any severe adverse events. Objective monitoring via fitness watches recorded marked increases in physical activity, as measured by active hours and calories burned both during the intervention compared to baseline. Preliminary efficacy was evaluated using the following PROs: European Organization for Research and Treatment of Cancer Quality of Life Questionnaire Core 30 (EORTC QLQ-C30), World Health Organization-5 scale (WHO-5), Allgemeine Selbstwirksamkeit Kurzskala (ASKU), Hospital Anxiety and Depression Scale questionnaire (HADS), and the distress thermometer. Descriptive analyses, as well as the respective median and range for each PROs at multiple timepoints (prior, during, past 1 month, and past 2 months), were collected. There was a strong signal in increased quality of life (as measured by self-reported survey: WHO-5 and ASKU) and a decrease in distress (as measured by Hospital Anxiety and Depression Scale questionnaire-Anxiety, Hospital Anxiety and Depression Scale questionnaire-Depression, and distress thermometer) during the intervention period and partly afterward in patients. Similar to other pilot or feasibility studies, this trial is limited by selection bias due to selecting for motivated subjects who were interested in skiing, as well as sampling bias due to its small sample size. Caregivers also benefited from decreased distress levels by participating in the intervention. This was the first ski-based dyadic exercise intervention in primary brain tumor patient–caregiver dyads and demonstrated that even moderately intense and technically demanding physical activity can be safely administered in this vulnerable population together with their caregivers.

There is a growing movement to characterize more traditional exercise methodologies as an intervention for PBT dyads. Halkett and colleagues published a qualitative study in 2021 exploring the experiences of glioblastoma (WHO grade 4) patients and their caregivers in a supervised and individualized exercise program during concurrent chemoradiotherapy [49]. The exercise intervention focused on the patient with the option for the caregiver to participate. Nineteen patients and fifteen caregivers engaged in twice-weekly sessions consisting of moderate-intensity aerobic and resistance training delivered at the treatment hospital. Through semi-structured interviews and thematic analysis, several perceived benefits emerged: participants valued the tailored nature of the program, reporting improvement in overall health, a reinstated sense of agency, opportunities for social interaction, and the maintenance of physical activity even beyond the direct treatment benefits. Caregivers also acknowledged secondary advantages, indicating that the intervention positively impacted their own well-being. However, challenges were also identified; these included managing the symptom burden of glioblastoma and its treatment while balancing the rigorous schedule demands of chemoradiation. Overall, the findings suggest that supervised exercise during chemoradiotherapy is perceived as feasible, safe, and beneficial by both patients and caregivers, while also highlighting logistical and symptomatic barriers that future trials will need to address. The authors also concluded that a quantitative pilot study evaluating a supervised moderate-intensity resistance-based exercise program for PBT patient–caregiver dyads is needed.

Martin and colleagues published a protocol for an intervention to introduce 150 min of virtual moderate aerobic and resistance training for 8 weeks [50]. The exercises, which are catered to individuals who are 8-weeks post completing their first round of treatment, will be split across two supervised and two unsupervised sessions, each aiming for an RPE moderate degree of intensity while utilizing a variety of equipment (e.g., resistance bands) provided by the organizers. In addition to measuring more established metrics of patient and caregiver wellbeing, such as EORTC QLQ-C30, EORTC QLQ-BN20, CQOLC, and DASS-21, this trial seeks to characterize functional fitness and sleep quality. Fitness was measured using movement-based assessments, such as a five-repetition maximum leg press and a 6-min walking test, whereas sleep quality will be measured using the Pittsburgh Sleep Quality Index, Insomnia Severity Index, an Oura Ring, and a sleep diary. If this study is successful, it will help to describe how exercise can impact not only patient and caregiver wellbeing, but also other markers of physiological health.

In 2025, Daun and colleagues published a qualitative analysis of how participants in the larger ACE-Neuro exercise trial, reflecting on how exercise contributed to their experience with PBTs [51]. Participants in the ACE-Neuro trial, a program in which patients attended two 30–60-min supervised workout circuits focused on resistance, aerobic exercise, and balance training per week for 12 weeks, were given the option to participate in a supplementary qualitative interview about their experience [52]. These interviews, which were offered to patients and their caregivers, followed a semi-structured format that included presenting interviewees with photographs of events that had transpired over the course of the program or other related concepts as a means of inspiring guided reflections. From these interviews, the organizers distilled participants’ stories into a central narrative: recognizing and adapting to their diagnosis, developing a new relationship with structured exercise as part of the trial, finding larger significance in the exercise, and learning to best adapt how they utilized exercise to their personal needs. Although the intervention itself was not caregiver focused, interviewees from both groups overall spoke highly of the program, demonstrating a positive opinion toward the opportunity to exercise and an appreciation for the associated benefits. Simliar to other previously mentioned largely descriptive studies, this work is susceptible to bias, such as reporting bias and observer bias.

## 4. Discussion

This scoping review identified eight publications that focused on exercise oncology in the PBT patient–caregiver dyad. As an emerging field of research, most of the identified publications were early-stage qualitative, pilot or feasibility studies, with one planned large multisite randomized clinical trial. The eight studies included studies comprising 5–67 dyads, with four being single-arm feasibility studies. Limitations included inadequate statistical power to detect clinically meaningful effects, a lack of randomization or appropriate control groups in some cases, and the possibility of selection bias, given the reliance on highly motivated patient–caregiver dyads who are willing to participate in intensive interventions. In addition, outcomes may be influenced by reporting bias, as many studies rely heavily on self-reported measures, such as Hawthorne bias, without corroborating objective endpoints. Furthermore, most of the included studies are early feasibility trials, which are not sufficiently powered for survival analysis or efficacy. However, these studies have shown that even moderately intense and technically complex physical activity can be delivered safely to this study population when undertaken with their caregivers, despite previous concerns for seizures and falls due to the presence of a primary brain tumor. Exercise programs implemented during chemoradiation tend to focus on utilizing exercise as a tool to help alleviate treatment-related adverse events, mainly fatigue, for which exercise is a well-known prescription ^12^. In contrast, exercise programs delivered following adjuvant chemotherapy tend to focus on improving the patient–caregiver dyad’s quality of life and enhance physical and psychological well-being as part of survivorship [29,49,53]. Videoconference is also a practical method of delivery for dyadic exercise intervention, and dyadic exercise may lead to higher adherence and retention than individual interventions in the PBT patient–caregiver dyad population.

Multiple meta-analyses have previously demonstrated that exercise can improve cancer-related fatigue and quality of life, especially for patients with solid tumors during and post cancer directed therapy [12,54]. This can be explained through multiple mechanisms such as improving mitochondrial function, neuroendocrine regulation, improving psychological distress through the mind–body connection, and improving cardiovascular efficiency and increasing oxygen delivery [53,55,56,57,58,59,60]. There is also emerging evidence that suggests that the benefits of exercise in patients with PBT may be mediated through modulation of the glymphatic system. The glymphatic system is a recently described waste clearance system for the brain that is implicated in disorders of cognition and aging [61,62]. However, primary brain tumors can also reduce glymphatic waste clearance, causing the accumulation of toxic waste solutes and pro-inflammatory signaling, leading to worsening quality of life and potentially even tumor progression [63,64]. Exercise has been shown to increase CSF influx and may mediate the glymphatic system in mice [58]. These findings highlight the need for future studies examining exercise-induced modulation of the glymphatic system in primary brain tumor patients. Additionally, growing evidence suggests that exercise may influence the primary brain tumor microenvironment and confer benefits through multiple immunologic mechanisms, including modulating the immune system and enhancing tumor immune surveillance [65]. Preclinical studies have also demonstrated the benefit of exercise in cancer immunotherapy by modulating microbiome metabolites [66].

Caregivers are at heightened risk of their own fatigue, social isolation, relationship strain, and adverse health outcomes, including higher rates of hypertension, cardiovascular disease, obesity, and mortality compared to non-caregivers [4,5,6,67]. Although the National Comprehensive Cancer Network (NCCN) recommends physical activity as a behavioral intervention for cancer-related fatigue, no evidence-based exercise protocols currently exist for the PBT patient–caregiver dyad [68]. Since the patient–caregiver dyad in PBT is an interdependent team, dyadic supportive care may offer greater benefits than patient or caregiver exercise alongside one another without dyadic interaction [35,38,69]. Such an intervention could foster teamwork and intimacy, enhance caregiver resilience, and potentially improve treatment adherence and patient survival. Moreover, by addressing functional decline and caregiver burnout, these programs could reduce preventable hospitalizations and treatment discontinuations, thereby alleviating some of the economic burden associated with PBT care. Most of the included studies included only patients with high-grade glioma (classified as WHO grade 3–4). This is possibly due to some of the studies focusing on exercise interventions during chemoradiation, naturally favoring high-grade PBT, as low-grade PBT might be treated with active surveillance rather than upfront chemoradiation [8]. Since high-grade PBT, particularly GBM, is the most common and aggressive form of primary malignant brain tumors, most current literature focuses on this disease due to the pressing need of this patient population [1].

Whenever possible, clinicians should attempt to include the caregivers during discussions with patients regarding the benefits of exercise in patients with PBT. The dose and description of the included structured exercise programs for PBT patient–caregiver dyads are presented in Table 2 and can be used as a framework for future exercise prescriptions. Engaging the patient and caregiver together in dyadic exercise may foster stronger support, adherence, and potentially can lead to better outcomes than simply patients and caregivers exercising alongside each other. Future large-scale quantitative trials with sufficient power to assess efficacy are needed to provide definitive answers. Although patient-reported outcomes are important to evaluate efficacy for both patients and caregivers, objective measurements such as biomarkers for mitochondrial functions and advanced imaging techniques are emerging as complementary tools for future clinical trials. Future studies should also include overall survival and progression-free survival as endpoints while considering age, MGMT status (which is a predictive biomarker for patients with glioblastoma), the type of exercise programs (dyadic versus individual interventions), and postoperative residual tumor volume^3^. Moreover, potential effect modifiers such as performance status and tumor grade (WHO grade 1–4) should also be included in planned subgroup analyses to determine whether dyadic exercise is an independent predictor of survival. As caregiver research is a rapidly growing field within neuro-oncology, other components of caregiver programs, such as dedicated psychosocial support, caregiver navigation, and bereavement support, should also be studied alongside exercise oncology interventions and their potential complimentary effects.

## 5. Conclusions

Current literature on dyadic exercise interventions in neuro-oncology consists primarily of small-scale feasibility and pilot studies. Initial findings have demonstrated that such interventions are safe and feasible. However, preliminary efficacy remains limited due to the risk of bias and the lack of statistical power. Larger randomized trials with objective endpoints are needed to define efficacy and guide evidence-based protocols. Of note, the identified studies focused on patients with a component of dyadic intervention, whereas caregivers were included mainly as participants rather than as the focus of dyadic interventions designed to strengthen the patient–caregiver relationship. Future trials looking at programs focused on the dyadic component of exercise to enhance communication, foster teamwork, and improve physical and mental wellbeing within the PBT patient–caregiver dyad are warranted. Importantly, given the preliminary findings from Milbury and colleagues that caregiver-focused interventions may confer greater benefits for caregivers than dyadic interventions by providing respite, future studies examining caregiver quality of life outside the dyadic context should also be encouraged [46].

## Figures and Tables

**Table 1:** Characteristics of the studies included in this scoping review.

Study	Participants	Clinical Characteristics *	Intervention	Objectives	Measures	Questionnaire Categories	Result
Milbury et al., 2018 [44] **Design:** single-arm pilot trial	***N* =** 5 dyads **Patient =** 80% Female **Patient Mean Age =** 52 **Caregiver** = 80% Female **Caregiver Mean Age** = 58 **Relationship:** Spouse (60%)	High-grade glioma (WHO grade 3–4) Treated with a minimum of 4 weeks of radiation therapy	Dyads attended 2 or 3 weekly sessions (60 min each) for 12 total sessions of dyadic yoga during the patient’s 5 to 6 weeks of radiation therapy.	Feasibility data and assessment of levels of cancer-related symptoms	Consent and retention rate **Questionnaires:** (1) BFI (2) CES-D (3) MDASI (4) SF-36 (5) PSQI	(1) fatigue (2) psychological distress (3) symptom burden (4) quality of life (5) sleep quality	83% consent 100% retention Clinically meaningful decrease in patient’s cancer symptoms. There was a reduction in patients’ sleep disturbance and an improvement in patient and caregiver’s mental quality of life.
Milbury et al., 2019 [45] **Design:** pilot randomized controlled trial	***N* =** 20 dyads **Patient =** 50% Female **Patient Mean Age =** 46 **Caregiver** = 70% Female **Caregiver Mean Age** = 50 **Relationship:** Spouse (50%)	PBT (WHO grade 1–4) Treated with a minimum of 20 fractions of radiation therapy	Dyads attended 2 or 3 weekly sessions (45 min each) for 12 total sessions of dyadic yoga during radiation therapy compared to the waitlist control group.	Feasibility data and assessment of preliminary efficacy	Consent, adherence, and retention **Questionnaires:** (1) BFI (2) CES-D (3) MDASI-BT (4) SF-36	(1) fatigue (2) psychological distress (3) symptom burden (brain tumor) (4) quality of life	70% consent 88% adherence 96% retention Clinically significant improvement in patient’s quality of life in the dyadic group compared to the waitlist control
Milbury et al., 2023 [46] **Design:** 3-arm randomized controlled trial	***N* =** 67 dyads **Patient =** 37% Female **Patient Mean Age =** 48 **Caregiver** = 79% Female **Caregiver Mean Age** = 52 **Relationship:** Spouse (69%)	PBT (WHO grade 1–4) Treated with a minimum of 5 weeks of radiation therapy	Dyads attended 45-min, 2–3 weekly sessions of yoga during radiation therapy for a total of 15 sessions in the dyadic intervention group vs. the individual caregiver group vs. usual care.	Feasibility and preliminary efficacy of the DY and CY interventions	Consent, adherence, and retention, as well as qualitative interview **Questionnaires:** (1) CES-D (2) SF-36 MCS (3) SF-36 PCS (4) CRA (Caregivers only)	(1) psychological distress (2) quality of life (mental component) (3) quality of life (physical component) (4) Caregiver reaction	59% consent 73% adherence 63% retention Caregivers had more perceived benefits in the individual caregiver yoga delivery arm compared to the dyadic intervention.
Rabe et al. 2024 [47] **Design:** study protocol for a multicenter randomized controlled trial	***N* =** 70 dyads (expected)	High-grade glioma (WHO grade 3–4)	Dyads attended 1 h per week of yoga delivered via video conference over 8 weeks compared to the waitlist control group.	Primary: to detect change in self-reported generalized anxiety at the end of the intervention Secondary: detect change in self-reported fear of progression and quality of life, as well as change in the biomarkers of stress	**Questionnaires** Primary: (1) GAD-7 Secondary: (2) FoP-Q-SF (3) PHQ-9 (4) EORTC QLQ-C30 **Biomarker** Brain-derived neurotropic factors Dehydroepiandrosterone/dehydroepiandrosterone sulfate Ferritin Hair cortisol	(1) psychological distress (2) psychological distress (3) psychological distress (4) quality of life	N.L.
Troschel et al., 2020 [48] **Design:** single-arm pilot trial	***N* =** 9 patients and 6 caregivers **Patient =** 44% Female **Patient Mean Age =** 47 **Caregiver** =50% Female **Caregiver Mean Age** = 49 **Relationship:** Spouse or relatives	PBT (WHO grade 1–4)	A full week ski-based exercise intervention for PBT patient–caregiver dyads	To describe the feasibility, safety, quality of life, and physical activity before and after the ski intervention in PBT patient–caregiver dyads.	Feasibility and safety Physical activity was tracked using fitness watches (both patients and caregivers) **Questionnaires:** (1) EORTC QLQ-C30 (2) WHO-5 (3) ASKU (4) HADS-A (5) HADS-D	(1) quality of life (2) psychological distress (3) psychological well-being (4) psychological distress (anxiety) (5) psychological distress (depression)	All participants completed the study without any severe adverse event. There was a strong increase in quantified physical activity, as well as quality of life (as measured using the questionnaire) during the intervention and shortly after.
Halkett et al., 2021 [49] **Design:** pilot qualitative study	***N* =** 19 patients, 15 caregivers	Glioblastoma (WHO grade 4) undergoing chemoradiation therapy	Combination of aerobic and resistance exercise during three 1-h sessions per week for 7 weeks during chemoradiation therapy for the patient with optional caregiver participation.	To describe the glioblastoma patient–caregiver dyad experience of participating in a tailored exercise intervention during chemoradiotherapy.	Semi-structured interviews and thematic analysis	N.L.	Glioblastoma patient–caregiver dyads expressed positive perceptions and experiences of a tailored exercise intervention during chemoradiotherapy.
Martin et al., 2024 [50] **Design:** single-arm intervention	N.L.	8-weeks post the completion of initial treatment for PBT	8-week virtual individualized exercise program targeting 150 min of RPE moderate-intensity resistance and aerobic exercise	Feasibility, acceptability, and safety of the intervention and efficacy of the intervention to improve mental, physical, and sleep health in the participants.	Feasibility, acceptability, safety, and semi-structured qualitative interviews Sleep measured via sleep quality indices, Oura Ring, and diary. Physical activity measured via functional fitness tests (e.g., 5RM leg press, 6-min walk test, etc.) **Questionnaires:** (1) EORTC QLQ-C30 (2) EORTC QLQ-BN20 (3) CQOLC (caregiver only) (4) DASS-21	(1) quality of life (2) quality of life (brain tumor) (3) caregiver burden (4) psychological distress	N.L.
Daun et al., 2025 [51] **Design:** single-arm intervention	*N* = 27 patients, 9 caregivers **Collective =** 57.1% Female **Collective Mean Age =** 51.4	Malignant or benign PBT	12-week virtual, in person, or hybrid individually tailored exercise program, including 1 on 1 sessions, group sessions, and health coaching focused on the patient	Qualitatively characterized the role of the intervention in the dyads’ experience with PBTs.	Consent, adherence, and qualitative interviews motivated by memory-eliciting photos	N.L.	54.9% consent (participation in supplemental interviews) 89.7% adherence Participants generally reflected positively on the opportunity to exercise, felt as though they benefited from exercising, and perceive exercise as a sustainable intervention for future patients.

* Criteria did not include generic logistics and willingness/consent, local language abilities, and ≥18, unless specified otherwise. N.L.: Not Listed.

**Table 2:** Dose and description for the prescribed exercise programs included in this scoping review.

Study	Intervention Timeline	Intervention Dose	Exercise Description	Exercise Type	Dyadic Component
Milbury et al., 2018 [44]	Concurrent with chemoradiotherapy	2–3 weekly 60 min sessions for 5–6 weeks (duration of radiotherapy), along with take home materials (pamphlet and DVD), enabling volunteer home practice.	In-person yoga sessions led by a certified instructor focusing on flexibility and deep relaxation.	Yoga intervention focusing on relaxation, mindfulness, flexibility, and stability	Yes. Dyadic component with partnered support and postures.
Milbury et al., 2019 [45]	Concurrent with chemoradiotherapy	2–3 weekly 45 min sessions totaling 12 sessions, along with take home materials (pamphlet and DVD), enabling volunteer home practice.	In-person yoga sessions led by a certified instructor focusing on flexibility and deep relaxation with accommodation for physical limitations.	Yoga intervention focusing on relaxation, mindfulness, flexibility, and stability	Yes. Dyadic component with partnered support and postures.
Milbury et al., 2023 [46]	Concurrent with chemoradiotherapy	2–3 weekly 45 min sessions totaling 15 sessions.	Both intervention groups included 4 in-person sessions, followed by 11 in-person or virtual sessions. All sessions were virtual after COVID. All sessions were led by certified yoga therapists. Dyadic yoga sessions focused on flexibility, relaxation, and meditation, with a focus on dyad communal coping and interconnectedness. Caregiver yoga sessions included the same content and structure without the focus on dyad dynamics.	Yoga intervention focusing on relaxation, mindfulness, flexibility, and stability	Dyadic yoga: Yes. Dyadic component with partnered support and postures. Caregiver yoga: No. Participation in class as a student, paying attention only to themselves.
Rabe et al., 2024 [47]	Anytime during treatment (recommended no earlier than 6 weeks post-surgery)	Once weekly 60 min session for 8 weeks, totaling 8 sessions.	Virtual Hatha Yoga sessions focusing on breathing exercises, physical poses and meditation, which are repeated each session. Sessions are limited to 10 participants. Patients and caregivers have dedicated sessions and are not in class together.	Yoga intervention focusing on physical activity, mindfulness, and breathing exercises	Yes. Partnered yoga intervention.
Troschel et al., 2020 [48]	Intervention was not specific to a treatment period so long as participant met inclusion criteria	Two 2-h skiing sessions per day for 7 days.	Skiing session involved instructor-led exercises and practice. Sessions were divided by skill level and interest, resulting in small groups (2–5 participants). Participants stayed the duration of the intervention at a nearby cabin with their families; food was provided.	Ski-based aerobic exercise	No. Patients and caregiver exercise alongside each other but no dyadic interaction.
Halkett et al., 2021 [49]	Concurrent with chemoradiotherapy	One weekly 60 min session for 7 weeks, totaling 7 sessions.	20–30 min of moderate to vigorous intensity cardiovascular exercise and resistance exercise incorporating 6–8 targeted exercises. Participants encouraged to progress toward a total of 150 min of moderate-intensity aerobic exercise weekly.	Aerobic and resistance exercise	No. Patients and caregivers exercise alongside each other but no dyadic interaction. Caregiver participation is optional.
Martin et al., 2024 [50]	Minimum 8 weeks following the initial course of treatment	Two supervised 45–60 min virtual sessions and two unsupervised, but defined 15–30 min sessions, totaling ~150 min per week for 8 weeks.	Supervised sessions: combination of resistance and aerobic training at RPE 12-14/20 Unsupervised sessions: aerobic training at RPE 12-14/20	Resistance and aerobic exercises, including resistance bands, kettlebells, and mat-based exercises (all of which are provided to the participants)	Patients and caregivers can choose to participate in a dyad or as individuals.
Daun et al., 2025 [51]	Any time before, during, or after treatment	Two weekly 30–60 min sessions (first 1 on 1 with an optional group substitute after wk 3), as well as 15–30 min health coaching session for 12 weeks.	Instructor-led exercise programs, including a warm up, main circuit, and cool down.	Circuit of RPE ~1–6 aerobic, resistance, and balance exercises	Caregivers included as observers but did not participate in exercise sessions.

## Data Availability

No new data were created or analyzed in this study.

## Supplementary Materials

The following supporting information can be downloaded at: https://www.mdpi.com/article/10.3390/curroncol33040193/s1, Supplemental File S1: search strategy.

## Abbreviations

BFI	Brief Fatigue Inventory
DT	Distress Thermometer
CES-D	Center for Epidemiological Studies—Depression measures
MDASI	MD Anderson Symptom Inventory
MDASI-BT	MD Anderson Symptom Inventory—Brain Tumor
SF-36	Study 36-item Short Form Survey
MCS	SF-36 domains for mental composite summary score
PCS	SF-36 domains for physical composite summary score
PSQI	Pittsburgh Sleep Quality Index
CRA	Caregiver Reaction Assessment
WHO-5	World Health Organization—5 scale
ASKU	Allgemeine Selbstwirksamkeit Kurzskala
HADS-A	Hospital Anxiety and Depression Scale questionnaire—anxiety
HADS-D	Hospital Anxiety and Depression Scale questionnaire—depression
FoP-Q-SF	Fear of Progression Questionnaire—Short Form
PHQ-9	Patient Health Questionnaire-9
EORTC QLQ-C30	European Organization for Research and Treatment of Cancer Quality of Life Questionnaire Core 30
CQOLC	The Caregiver Quality of Life Index—Cancer (CQOLC) scale
DASS-21	Depression, Anxiety, and Stress Scale
