# Supplementary material for: A Scoping Review of Exercise Oncology in the Primary Brain Tumor Patient–Caregiver Dyad
Source: Curr Oncol. 2026 Mar 30;33(4):193. doi: 10.3390/curroncol33040193 (PMC13114407; doi:10.3390/curroncol33040193)
Supplement: Supplementary file 1 [file curroncol-33-00193-s001.zip › curroncol-4161401-supplementary.pdf]

MEDLINE (PubMed)

Search number	Query	Results
23	#22 NOT ((infant[mesh] OR child[mesh] OR adolescent[mesh]) NOT adult[mesh])	236
22	#5 AND #16 AND #21	263
21	#17 OR #18 OR #19 OR #20	1,352,058
20	caregiver*[tiab] OR carer*[tiab] OR "care partner*"[tiab] OR "support person*"[tiab] OR "informal care*"[tiab] OR "primary caregiver*"[tiab] OR "co-survivor*"[tiab] OR "significant other*"[tiab] OR "next of kin"[tiab] OR "loved one*"[tiab] OR "caregiving dyad"[tiab] OR "dyadic relationship*"[tiab] OR "patient-caregiver"[tiab] OR "caregiver-patient"[tiab] OR partner*[tiab] OR spouse*[tiab] OR couple*[tiab] OR dyad*[tiab] OR "couple-based"[tiab] OR "couples-based"[tiab] OR "dyadic"[tiab] OR "partner-based"[tiab] OR "family member*"[tiab]	1,029,888
19	"Family"[Mesh]	397,428
18	"Spouses"[Mesh]	12,565
17	"Caregivers"[Mesh]	60,176
16	#6 OR #7 OR #8 OR #9 OR #10 OR #11 OR #12 OR #13 OR #14 OR #15	1,577,184
15	exercis*[tiab] OR "physical activit*"[tiab] OR "physical therap*"[tiab] OR "physiotherapy"[tiab] OR rehab*[tiab] OR "prehab*"[tiab] OR "kinesiotherapy"[tiab] OR "sports"[tiab] OR "aerobic*"[tiab] OR "weight lifting"[tiab] OR "HIIT"[tiab] OR "muscle strength"[tiab] OR "fitness"[tiab] OR "walking"[tiab] OR "running"[tiab] OR "jogging"[tiab] OR "cycling"[tiab] OR "bicycling"[tiab] OR "swimming"[tiab] OR "yoga"[tiab] OR "pilates"[tiab] OR "tai chi"[tiab] OR "qigong"[tiab] OR "dance"[tiab] OR "dancing"[tiab] OR "active lifestyle"[tiab] OR "flexibility"[tiab] OR "stretching"[tiab] OR "resistance training"[tiab:~3] OR "strength training"[tiab:~3] OR "endurance training"[tiab:~3] OR "balance training"[tiab:~3] OR "high intensity interval training"[tiab]	1,471,365
14	"Dance Therapy"[Mesh]	545
13	"Qigong"[Mesh]	460
12	"Tai Ji"[Mesh]	1,742
11	"Yoga"[Mesh]	4,495
10	"Recreation Therapy"[Mesh]	154
9	"Sports"[Mesh]	237,957
8	"Physical Fitness"[Mesh]	40,092
7	"Exercise Therapy"[Mesh]	73,591
6	"Exercise"[Mesh]	280,618
5	#1 OR #2 OR #3 OR #4	355,563
4	(("brain"[Title/Abstract] OR "cerebral"[Title/Abstract] OR "intracranial"[Title/Abstract] OR "intracerebral"[Title/Abstract] OR	232,070

	"CNS"[Title/Abstract] OR "central nervous system"[Title/Abstract]) AND ("tumor*"[Title/Abstract] OR "tumour*"[Title/Abstract] OR "cancer*"[Title/Abstract] OR "neoplasm*"[Title/Abstract] OR "malignan*"[Title/Abstract] OR "carcinoma*"[Title/Abstract] OR "glioma*"[Title/Abstract] OR "glioblastoma*"[Title/Abstract] OR "astrocytoma*"[Title/Abstract] OR "oligodendroglioma*"[Title/Abstract] OR "ependymoma*"[Title/Abstract] OR "medulloblastoma*"[Title/Abstract]))	
3	"Central Nervous System Neoplasms"[Mesh:NoExp]	9,031
2	"Glioma"[MeSH Terms]	109,767
1	"Brain Neoplasms"[MeSH Terms]	186,530

Embase (Elsevier)

No.	Query	Results
#20	#19 NOT (([infant]/lim OR [child]/lim OR [adolescent]/lim) NOT [adult]/lim)	794
#19	#5 AND #14 AND #18	969
#18	#15 OR #16 OR #17	1886836
#17	caregiver*:ti,ab,kw OR carer*:ti,ab,kw OR 'care partner*':ti,ab,kw OR 'support person*':ti,ab,kw OR 'informal care*':ti,ab,kw OR 'primary caregiver*':ti,ab,kw OR 'co-survivor*':ti,ab,kw OR 'co survivor*':ti,ab,kw OR 'significant other*':ti,ab,kw OR 'next of kin':ti,ab,kw OR 'loved one*':ti,ab,kw OR 'caregiving dyad':ti,ab,kw OR 'dyadic relationship*':ti,ab,kw OR 'patient-caregiver':ti,ab,kw OR 'patient caregiver':ti,ab,kw OR 'caregiver-patient':ti,ab,kw OR 'caregiver patient':ti,ab,kw OR partner*:ti,ab,kw OR spouse*:ti,ab,kw OR couple*:ti,ab,kw OR dyad*:ti,ab,kw OR 'couple-based':ti,ab,kw OR 'couple based':ti,ab,kw OR 'couples-based':ti,ab,kw OR 'couples based':ti,ab,kw OR dyadic:ti,ab,kw OR 'partner-based':ti,ab,kw OR 'partner based':ti,ab,kw OR 'family member*':ti,ab,kw	1296275
#16	family'/exp	700271
#15	caregiver'/exp	157602
#14	#6 OR #7 OR #8 OR #9 OR #10 OR #11 OR #12 OR #13	2154891
#13	((resistance OR strength OR endurance OR balance) NEAR/3 training):ti,ab,kw	47453
#12	exercis*:ti,ab,kw OR 'physical activit*':ti,ab,kw OR 'physical therap*':ti,ab,kw OR physiotherapy:ti,ab,kw OR rehab*:ti,ab,kw OR prehab*:ti,ab,kw OR kinesiotherapy:ti,ab,kw OR sports:ti,ab,kw OR aerobic*:ti,ab,kw OR 'weight lifting':ti,ab,kw OR hiit:ti,ab,kw OR 'muscle strength':ti,ab,kw OR fitness:ti,ab,kw OR walking:ti,ab,kw OR running:ti,ab,kw OR jogging:ti,ab,kw OR cycling:ti,ab,kw OR bicycling:ti,ab,kw OR swimming:ti,ab,kw OR yoga:ti,ab,kw OR pilates:ti,ab,kw OR 'tai chi':ti,ab,kw OR qigong:ti,ab,kw OR dance:ti,ab,kw OR dancing:ti,ab,kw OR 'active lifestyle':ti,ab,kw OR flexibility:ti,ab,kw OR stretching:ti,ab,kw OR 'high intensity interval training':ti,ab,kw	1943275

#11	dance therapy'/de	1127
#10	recreational therapy'/de	796
#9	sport'/exp	257875
#8	fitness'/de	54177
#7	kinesiotherapy'/exp	130397
#6	exercise'/exp	560174
#5	#1 OR #2 OR #3 OR #4	605474
#4	(brain:ti,ab,kw OR cerebral:ti,ab,kw OR intracranial:ti,ab,kw OR intracerebral:ti,ab,kw OR 'cns':ti,ab,kw OR 'central nervous system':ti,ab,kw) AND (tumor*:ti,ab,kw OR tumour*:ti,ab,kw OR cancer*:ti,ab,kw OR neoplasm*:ti,ab,kw OR malignan*:ti,ab,kw OR carcinoma*:ti,ab,kw OR glioma*:ti,ab,kw OR glioblastoma*:ti,ab,kw OR astrocytoma*:ti,ab,kw OR oligodendroglioma*:ti,ab,kw OR ependymoma*:ti,ab,kw OR medulloblastoma*:ti,ab,kw)	361561
#3	central nervous system tumor'/de	15524
#2	glioma'/exp	214660
#1	brain tumor'/exp	279169

CINAHL (EBSCO)

S#	Query (user-entered)	Results (count)
S22	S5 AND S17 AND S21	87
S21	S18 OR S19 OR S20	531478
S20	(TI "caregiver*" OR AB "caregiver*" OR TI "carer*" OR AB "carer*" OR TI "care partner*" OR AB "care partner*" OR TI "support person*" OR AB "support person*" OR TI "informal care*" OR AB "informal care*" OR TI "primary caregiver*" OR AB "primary caregiver*" OR TI "co-survivor*" OR AB "co-survivor*" OR TI "significant other*" OR AB "significant other*" OR TI "next of kin" OR AB "next of kin" OR TI "loved one*" OR AB "loved one*" OR TI "caregiving dyad" OR AB "caregiving dyad" OR TI "dyadic relationship*" OR AB "dyadic relationship*" OR TI "patient-caregiver" OR AB "patient-caregiver" OR TI "caregiver-patient" OR AB "caregiver-patient" OR TI "partner*" OR AB "partner*" OR TI "spouse*" OR AB "spouse*" OR TI "couple*" OR AB "couple*" OR TI "dyad*" OR AB "dyad*" OR TI "couple-based" OR AB "couple-based" OR TI "couples-based" OR AB "couples-based" OR TI "dyadic" OR AB "dyadic" OR TI "partner-based" OR AB "partner-based" OR TI "family member*" OR AB "family member*")	278580
S19	MH "Family+"	310241
S18	MH "Caregivers"	51240
S17	S6 OR S7 OR S8 OR S9 OR S10 OR S11 OR S12 OR S13 OR S14 OR S15 OR S16	600825
S16	(TI (resistance N3 training) OR AB (resistance N3 training) OR TI (strength N3 training) OR AB (strength N3 training) OR TI (endurance N3 training) OR AB (endurance N3 training) OR TI (balance N3 training) OR AB (balance N3 training))	16321
S15	(TI "exercis*" OR AB "exercis*" OR TI "physical activit*" OR AB "physical activit*" OR TI "physical therap*" OR AB "physical therap*" OR TI "physiotherapy" OR AB	494957

	"physiotherapy" OR TI "rehab*" OR AB "rehab*" OR TI "prehab*" OR AB "prehab*" OR TI "kinesiotherapy" OR AB "kinesiotherapy" OR TI "sports" OR AB "sports" OR TI "aerobic*" OR AB "aerobic*" OR TI "weight lifting" OR AB "weight lifting" OR TI "HIIT" OR AB "HIIT" OR TI "muscle strength" OR AB "muscle strength" OR TI "fitness" OR AB "fitness" OR TI "walking" OR AB "walking" OR TI "running" OR AB "running" OR TI "jogging" OR AB "jogging" OR TI "cycling" OR AB "cycling" OR TI "bicycling" OR AB "bicycling" OR TI "swimming" OR AB "swimming" OR TI "yoga" OR AB "yoga" OR TI "pilates" OR AB "pilates" OR TI "tai chi" OR AB "tai chi" OR TI "qigong" OR AB "qigong" OR TI "dance" OR AB "dance" OR TI "dancing" OR AB "dancing" OR TI "active lifestyle" OR AB "active lifestyle" OR TI "flexibility" OR AB "flexibility" OR TI "stretching" OR AB "stretching" OR TI "high intensity interval training" OR AB "high intensity interval training")	
S14	MH "Dance Therapy"	1252
S13	MH "Qigong"	942
S12	MH "Tai Chi"	2554
S11	MH "Yoga+"	10462
S10	MH "Recreational Therapy"	1930
S9	MH "Sports+"	100564
S8	MH "Physical Fitness+"	24313
S7	MH "Therapeutic Exercise+"	73370
S6	MH "Exercise+"	143138
S5	S1 OR S2 OR S3 OR S4	42463
S4	(TI "brain" OR AB "brain" OR TI "cerebral" OR AB "cerebral" OR TI "intracranial" OR AB "intracranial" OR TI "intracerebral" OR AB "intracerebral" OR TI "CNS" OR AB "CNS" OR TI "central nervous system" OR AB "central nervous system") AND (TI "tumor*" OR AB "tumor*" OR TI "tumour*" OR AB "tumour*" OR TI "cancer*" OR AB "cancer*" OR TI "neoplasm*" OR AB "neoplasm*" OR TI "malignan*" OR AB "malignan*" OR TI "carcinoma*" OR AB "carcinoma*" OR TI "glioma*" OR AB "glioma*" OR TI "glioblastoma*" OR AB "glioblastoma*" OR TI "astrocytoma*" OR AB "astrocytoma*" OR TI "oligodendroglioma*" OR AB "oligodendroglioma*" OR TI "ependymoma*" OR AB "ependymoma*" OR TI "medulloblastoma*" OR AB "medulloblastoma*")	27819
S3	MH "Central Nervous System Neoplasms"	2214
S2	MH "Glioma+"	11777
S1	MH "Brain Neoplasms+"	18875

Rehabilitation and Sports Medicine Source (EBSCO)

S#	Query (user-entered)	Results (count)
S13	S1 AND S9 AND S12	9
S12	S10 OR S11	14379
S11	(TI "caregiver*" OR AB "caregiver*" OR KW "caregiver*" OR TI "carer*" OR AB "carer*" OR KW "carer*" OR TI "partner*" OR AB "partner*" OR KW "partner*" OR TI "spouse*" OR AB "spouse*" OR KW "spouse*" OR TI "couple*" OR AB "couple*" OR KW "couple*" OR TI	14224

	"dyad*" OR AB "dyad*" OR KW "dyad*" OR TI "family member*" OR AB "family member*" OR KW "family member*" OR TI "support person*" OR AB "support person*" OR KW "support person*" OR TI "informal care*" OR AB "informal care*" OR KW "informal care*" OR TI "co-survivor*" OR AB "co-survivor*" OR KW "co-survivor*" OR TI "significant other*" OR AB "significant other*" OR KW "significant other*" OR TI "next of kin" OR AB "next of kin" OR KW "next of kin" OR TI "loved one*" OR AB "loved one*" OR KW "loved one*")	
S10	DE "CAREGIVERS"	1323
S9	S2 OR S3 OR S4 OR S5 OR S6 OR S7 OR S8	184376
S8	(TI (resistance N3 training) OR AB (resistance N3 training) OR KW (resistance N3 training) OR TI (strength N3 training) OR AB (strength N3 training) OR KW (strength N3 training) OR TI (endurance N3 training) OR AB (endurance N3 training) OR KW (endurance N3 training) OR TI (balance N3 training) OR AB (balance N3 training) OR KW (balance N3 training))	11411
S7	(TI "exercis*" OR AB "exercis*" OR KW "exercis*" OR TI "physical activit*" OR AB "physical activit*" OR KW "physical activit*" OR TI "physical therap*" OR AB "physical therap*" OR KW "physical therap*" OR TI "physiotherapy" OR AB "physiotherapy" OR KW "physiotherapy" OR TI "rehab*" OR AB "rehab*" OR KW "rehab*" OR TI "prehab*" OR AB "prehab*" OR KW "prehab*" OR TI "kinesiotherapy" OR AB "kinesiotherapy" OR KW "kinesiotherapy" OR TI "sports" OR AB "sports" OR KW "sports" OR TI "aerobic*" OR AB "aerobic*" OR KW "aerobic*" OR TI "weight lifting" OR AB "weight lifting" OR KW "weight lifting" OR TI "HIIT" OR AB "HIIT" OR KW "HIIT" OR TI "muscle strength" OR AB "muscle strength" OR KW "muscle strength" OR TI "fitness" OR AB "fitness" OR KW "fitness" OR TI "walking" OR AB "walking" OR KW "walking" OR TI "running" OR AB "running" OR KW "running" OR TI "jogging" OR AB "jogging" OR KW "jogging" OR TI "cycling" OR AB "cycling" OR KW "cycling" OR TI "bicycling" OR AB "bicycling" OR KW "bicycling" OR TI "swimming" OR AB "swimming" OR KW "swimming" OR TI "yoga" OR AB "yoga" OR KW "yoga" OR TI "pilates" OR AB "pilates" OR KW "pilates" OR TI "tai chi" OR AB "tai chi" OR KW "tai chi" OR TI "qigong" OR AB "qigong" OR KW "qigong" OR TI "dance" OR AB "dance" OR KW "dance" OR TI "dancing" OR AB "dancing" OR KW "dancing" OR TI "active lifestyle" OR AB "active lifestyle" OR KW "active lifestyle" OR TI "flexibility" OR AB "flexibility" OR KW "flexibility" OR TI "stretching" OR AB "stretching" OR KW "stretching" OR TI "high intensity interval training" OR AB "high intensity interval training" OR KW "high intensity interval training")	173506
S6	DE "DANCE therapy"	121
S5	DE "RECREATIONAL therapy"	505
S4	(DE "SPORTS" OR DE "AMATEUR sports" OR DE "AQUATIC sports" OR DE "BALL games" OR DE "BASEBALL" OR DE "COMBAT sports" OR DE "CONTACT sports" OR DE "CROSS-training (Sports)" OR DE "DISC golf" OR DE "ENDURANCE sports" OR DE "EXTREME sports" OR DE "GAELIC games" OR DE "GYMNASTICS" OR DE "HOCKEY" OR DE "INDIVIDUAL sports" OR DE "PARKOUR" OR DE "RACKET games" OR DE "RECREATIONAL sports" OR DE "ROLLER skating" OR DE "SHOOTING (Sports)" OR DE "SKATEBOARDING" OR DE "SOFTBALL" OR DE "TEAM sports" OR DE "TRACEURS" OR DE "WINTER sports")	11531
S3	(DE "PHYSICAL fitness" OR DE "ANAEROBIC exercises" OR DE "BODYBUILDING" OR DE "CIRCUIT training" OR DE "COMPOUND exercises" OR DE "ISOLATION exercises" OR DE "LIANGONG" OR DE "PERIODIZATION training")	13153

S2	(DE "EXERCISE" OR DE "ABDOMINAL exercises" OR DE "AEROBIC exercises" OR DE "ANAEROBIC exercises" OR DE "AQUATIC exercises" OR DE "ARM exercises" OR DE "BACK exercises" OR DE "BREATHING exercises" OR DE "BREEMA" OR DE "BUTTOCKS exercises" OR DE "CALISTHENICS" OR DE "CHAIR exercises" OR DE "CHEST exercises" OR DE "CIRCUIT training" OR DE "COMPOUND exercises" OR DE "COOLDOWN" OR DE "DO-in" OR DE "EXERCISE therapy" OR DE "EXERCISE video games" OR DE "FALUN gong exercises" OR DE "FOOT exercises" OR DE "GYMNASTICS" OR DE "HAND exercises" OR DE "HATHA yoga" OR DE "HIP exercises" OR DE "ISOKINETIC exercise" OR DE "ISOLATION exercises" OR DE "ISOMETRIC exercise" OR DE "ISOTONIC exercise" OR DE "KNEE exercises" OR DE "LEG exercises" OR DE "LIANGONG" OR DE "MULAN quan" OR DE "PILATES method" OR DE "PLYOMETRICS" OR DE "QI gong" OR DE "REDUCING exercises" OR DE "RUNNING" OR DE "SHOULDER exercises" OR DE "STRENGTH training" OR DE "STRESS management exercises" OR DE "TAI chi" OR DE "TREADMILL exercise" OR DE "WHEELCHAIR workouts" OR DE "YOGA")	45654
S1	((TI "brain" OR AB "brain" OR KW "brain" OR TI "cerebral" OR AB "cerebral" OR KW "cerebral" OR TI "intracranial" OR AB "intracranial" OR KW "intracranial" OR TI "intracerebral" OR AB "intracerebral" OR KW "intracerebral" OR TI "CNS" OR AB "CNS" OR KW "CNS" OR TI "central nervous system" OR AB "central nervous system" OR KW "central nervous system") AND (TI "tumor*" OR AB "tumor*" OR KW "tumor*" OR TI "tumour*" OR AB "tumour*" OR KW "tumour*" OR TI "cancer*" OR AB "cancer*" OR KW "cancer*" OR TI "neoplasm*" OR AB "neoplasm*" OR KW "neoplasm*" OR TI "malignan*" OR AB "malignan*" OR KW "malignan*" OR TI "carcinoma*" OR AB "carcinoma*" OR KW "carcinoma*" OR TI "glioma*" OR AB "glioma*" OR KW "glioma*" OR TI "glioblastoma*" OR AB "glioblastoma*" OR KW "glioblastoma*" OR TI "astrocytoma*" OR AB "astrocytoma*" OR KW "astrocytoma*" OR TI "oligodendroglioma*" OR AB "oligodendroglioma*" OR KW "oligodendroglioma*" OR TI "ependymoma*" OR AB "ependymoma*" OR KW "ependymoma*" OR TI "medulloblastoma*" OR AB "medulloblastoma*" OR KW "medulloblastoma*"))	764

Cochrane Central Register of Controlled Trials (Ovid)

1	exp Brain Neoplasms/	3217
2	exp Glioma/	2070
3	Central Nervous System Neoplasms/	278
4	((brain or cerebral or intracranial or intracerebral or CNS or "central nervous system") and (tumor* or tumour* or cancer* or neoplasm* or malignan* or carcinoma* or glioma* or glioblastoma* or astrocytoma* or oligodendroglioma* or ependymoma* or medulloblastoma*)).ti,ab,kw.	12483
5	1 or 2 or 3 or 4	14503
6	exp Exercise/	41259
7	exp Exercise Therapy/	23633
8	exp Physical Fitness/	5161
9	exp Sports/	23211
10	Recreation Therapy/	27

11	Yoga/	1269
12	Tai Ji/	624
13	Qigong/	188
14	Dance Therapy/	142
15	(exercis* or "physical activit*" or "physical therap*" or physiotherapy or rehab* or prehab* or kinesiotherapy or sports or aerobic* or "weight lifting" or HIIT or "muscle strength" or fitness or walking or running or jogging or cycling or bicycling or swimming or yoga or pilates or "tai chi" or qigong or dance or dancing or "active lifestyle" or flexibility or stretching or "high intensity interval training" or ((resistance or strength or endurance or balance) adj3 training)).ti,ab,kw.	27786<br>7
16	6 or 7 or 8 or 9 or 10 or 11 or 12 or 13 or 14 or 15	28260<br>8
17	Caregivers/	4210
18	Spouses/	531
19	exp Family/	15447
20	(caregiver* or carer* or partner* or spouse* or couple* or dyad* or "family member*" or "support person*" or "informal care*" or "co-survivor*" or "significant other*" or "next of kin" or "loved one*").ti,ab,kw.	65001
21	17 or 18 or 19 or 20	76051
22	5 and 16 and 21	95

ClinicalTrials.gov

Condition/Disease: "Brain Tumor" OR "Brain Neoplasm" OR "Brain Cancer" OR Glioma OR Glioblastoma OR Astrocytoma OR Oligodendroglioma OR Meningioma OR Ependymoma OR Medulloblastoma OR "Central Nervous System Tumor"

Other terms: Caregiver OR Carer OR "Care Partner" OR Partner OR Spouse OR Family OR Dyad OR "Support Person"

Intervention/treatment: Exercise OR "Physical Activity" OR "Physical Therapy" OR Physiotherapy OR Rehabilitation OR Yoga OR Pilates OR "Tai Chi" OR Qigong OR Training OR Sports OR Walking

Age: Adult (18 - 64), Older adult (65+)

WHO International Clinical Trials Registry Platform Search Portal

Condition: brain tumor OR brain cancer OR glioma OR glioblastoma OR astrocytoma OR CNS tumor OR central nervous system tumor

Intervention: exercise OR physical activity OR rehabilitation OR yoga OR pilates OR training OR sports
